# Supplementary material for: High-Resolution Mapping of Crossover and Non-crossover Recombination Events by Whole-Genome Re-sequencing of an Avian Pedigree
Source: PLoS Genet. 2016 May 24;12(5):e1006044. doi: 10.1371/journal.pgen.1006044 (PMC4878770; doi:10.1371/journal.pgen.1006044)
Supplement: S1 Table — (DOCX) [file pgen.1006044.s001.docx]

**Supplementary Table 1.**

| Individual (generation) | Depth of coverage |
| --- | --- |
| CJ4 (P) | 41.9 |
| CJ0 (P) | 43.2 |
| BL1 (P) | 42.1 |
| BY8 (P) | 42.3 |
| CL2 (F_1_) | 43.0 |
| CA2 (F_1_) | 39.9 |
| CS4 (F_2_) | 43.4 |
| CS5 (F_2_) | 36.9 |
| CS6 (F_2_) | 43.1 |
| CS7 (F_2_) | 45.3 |
| CS9 (F_2_) | 45.4 |
