# Supplementary material for: High-Resolution Mapping of Crossover and Non-crossover Recombination Events by Whole-Genome Re-sequencing of an Avian Pedigree
Source: PLoS Genet. 2016 May 24;12(5):e1006044. doi: 10.1371/journal.pgen.1006044 (PMC4878770; doi:10.1371/journal.pgen.1006044)
Supplement: S2 Table — (DOCX) [file pgen.1006044.s002.docx]

**Supplementary Table 2**.

| Chromosome | Position of upstream flanking marker | Position of downstream flanking marker |
| --- | --- | --- |
| Chr1 | 697103 | 699135 |
| Chr1 | 2522238 | 2523587 |
| Chr1 | 4129768 | 4130027 |
| Chr1 | 8926300 | 8928354 |
| Chr1 | 20109012 | 20109626 |
| Chr1 | 34601581 | 34602181 |
| Chr1 | 39787290 | 39788905 |
| Chr1 | 44685153 | 44685325 |
| Chr1 | 49521499 | 49522757 |
| Chr1 | 54690918 | 54691498 |
| Chr1 | 62248715 | 62249864 |
| Chr1 | 64699907 | 64701013 |
| Chr1 | 67404864 | 67406520 |
| Chr1 | 75463618 | 75464317 |
| Chr1 | 77655719 | 77657829 |
| Chr1 | 78273267 | 78274911 |
| Chr1 | 84814772 | 84816102 |
| Chr1 | 91005366 | 91006009 |
| Chr1 | 92479538 | 92506307 |
| Chr1 | 98666795 | 98667680 |
| Chr1 | 102873978 | 102874696 |
| Chr1 | 102879092 | 102879351 |
| Chr1 | 106388677 | 106391490 |
| Chr1 | 106823152 | 106824557 |
| Chr1 | 114335995 | 114336590 |
| Chr1 | 117919242 | 117920426 |
| Chr1A | 2403244 | 2404299 |
| Chr1A | 2732242 | 2732297 |
| Chr1A | 3489718 | 3492700 |
| Chr1A | 5027144 | 5028493 |
| Chr1A | 6550337 | 6551492 |
| Chr1A | 18763888 | 18767367 |
| Chr1A | 27794969 | 27796653 |
| Chr1A | 34152990 | 34165164 |
| Chr1A | 38999649 | 38999961 |
| Chr1A | 45614663 | 45619132 |
| Chr1A | 50900411 | 50904595 |
| Chr1A | 51151579 | 51152575 |
| Chr1A | 52205134 | 52205454 |
| Chr1A | 64563884 | 64563919 |
| Chr1A | 64955731 | 64957205 |
| Chr1A | 67862135 | 67862316 |
| Chr1A | 68654485 | 68655195 |
| Chr1A | 73940417 | 73943520 |
| Chr2 | 2767815 | 2769101 |
| Chr2 | 10473642 | 10479656 |
| Chr2 | 11095824 | 11098162 |
| Chr2 | 11615970 | 11622486 |
| Chr2 | 20284522 | 20286525 |
| Chr2 | 25340134 | 25340405 |
| Chr2 | 32031693 | 32031949 |
| Chr2 | 35985362 | 35989468 |
| Chr2 | 36012639 | 36013388 |
| Chr2 | 39177643 | 39179804 |
| Chr2 | 42503278 | 42504667 |
| Chr2 | 43471170 | 43472018 |
| Chr2 | 54784411 | 54788160 |
| Chr2 | 58434839 | 58439250 |
| Chr2 | 59837920 | 59838621 |
| Chr2 | 63377206 | 63378350 |
| Chr2 | 64387879 | 64388180 |
| Chr2 | 69300111 | 69301145 |
| Chr2 | 102166184 | 102167375 |
| Chr2 | 106312838 | 106314163 |
| Chr2 | 113061574 | 113061898 |
| Chr2 | 118464259 | 118465396 |
| Chr2 | 132228146 | 132228729 |
| Chr2 | 132814743 | 132818743 |
| Chr2 | 137630291 | 137632588 |
| Chr2 | 145397356 | 145398009 |
| Chr2 | 153432185 | 153432909 |
| Chr2 | 154633138 | 154635114 |
| Chr2 | 155290264 | 155290524 |
| Chr3 | 12086 | 14523 |
| Chr3 | 533933 | 534326 |
| Chr3 | 15320093 | 15320497 |
| Chr3 | 21911640 | 21912657 |
| Chr3 | 25678669 | 25681722 |
| Chr3 | 39154484 | 39155568 |
| Chr3 | 43871929 | 43872737 |
| Chr3 | 58203120 | 58203750 |
| Chr3 | 79948226 | 79948839 |
| Chr3 | 82496109 | 82502594 |
| Chr3 | 85221829 | 85222716 |
| Chr3 | 89494811 | 89496559 |
| Chr3 | 97441017 | 97441538 |
| Chr3 | 98900960 | 98901492 |
| Chr3 | 101073604 | 101073992 |
| Chr3 | 112978796 | 112979989 |
| Chr3 | 113165487 | 113166314 |
| Chr3 | 113487102 | 113488639 |
| Chr3 | 114179682 | 114181468 |
| Chr3 | 114481050 | 114496639 |
| Chr3 | 115554977 | 115557666 |
| Chr4 | 979723 | 979874 |
| Chr4 | 1397977 | 1555314 |
| Chr4 | 7732937 | 7739490 |
| Chr4 | 10657190 | 10717265 |
| Chr4 | 14837297 | 14840774 |
| Chr4 | 16687830 | 16689294 |
| Chr4 | 17648646 | 17666914 |
| Chr4 | 39758708 | 39760221 |
| Chr4 | 41861845 | 41867227 |
| Chr4 | 47088323 | 47088650 |
| Chr4 | 50891387 | 50903699 |
| Chr4 | 61209823 | 61210869 |
| Chr4 | 65388229 | 65395095 |
| Chr4 | 68744448 | 68745515 |
| Chr4 | 69682964 | 69685985 |
| Chr4 | 70080296 | 70080582 |
| Chr4A | 1132344 | 1133298 |
| Chr4A | 5667570 | 5668488 |
| Chr4A | 5954766 | 5955444 |
| Chr4A | 17746421 | 17863875 |
| Chr5 | 2976154 | 2976687 |
| Chr5 | 3343708 | 3343850 |
| Chr5 | 3746358 | 3750955 |
| Chr5 | 4415455 | 4534231 |
| Chr5 | 11128583 | 11131097 |
| Chr5 | 11474010 | 11474624 |
| Chr5 | 11664357 | 11665451 |
| Chr5 | 12353527 | 12366278 |
| Chr5 | 23227453 | 23227691 |
| Chr5 | 26556179 | 26558410 |
| Chr5 | 27400632 | 27403419 |
| Chr5 | 28700292 | 28700407 |
| Chr5 | 33224499 | 33226236 |
| Chr5 | 37885490 | 37887888 |
| Chr5 | 46864252 | 46866729 |
| Chr5 | 56535525 | 56535642 |
| Chr5 | 57823744 | 57926816 |
| Chr5 | 58106347 | 58106637 |
| Chr5 | 62263599 | 62266800 |
| Chr5 | 62412212 | 62413015 |
| Chr6 | 2269664 | 2270415 |
| Chr6 | 4966525 | 4968016 |
| Chr6 | 5987720 | 5992035 |
| Chr6 | 10844271 | 10844679 |
| Chr6 | 11484127 | 11484294 |
| Chr6 | 23817819 | 23823884 |
| Chr6 | 24093913 | 24097573 |
| Chr6 | 27561270 | 27564197 |
| Chr6 | 34528284 | 34529954 |
| Chr7 | 997704 | 1022639 |
| Chr7 | 7385152 | 7385701 |
| Chr7 | 7696091 | 7698567 |
| Chr7 | 12237986 | 12241017 |
| Chr7 | 16547651 | 16551213 |
| Chr7 | 22918409 | 22920029 |
| Chr7 | 32396866 | 32398908 |
| Chr7 | 38011187 | 38013488 |
| Chr7 | 38128757 | 38132420 |
| Chr7 | 38382220 | 38384110 |
| Chr8 | 2420639 | 2421354 |
| Chr8 | 6853817 | 6854343 |
| Chr8 | 7251381 | 7252818 |
| Chr8 | 15524747 | 15527542 |
| Chr8 | 18658568 | 18659181 |
| Chr8 | 27720161 | 27723953 |
| Chr8 | 27913287 | 27916193 |
| Chr8 | 28038491 | 28038862 |
| Chr8 | 30854261 | 30856970 |
| Chr8 | 30893586 | 30907354 |
| Chr9 | 177249 | 177478 |
| Chr9 | 1754493 | 1754781 |
| Chr9 | 5861478 | 5861785 |
| Chr9 | 20953459 | 20954082 |
| Chr9 | 24083919 | 24084212 |
| Chr9 | 24742412 | 24743842 |
| Chr10 | 1368279 | 1372146 |
| Chr10 | 1614685 | 1616792 |
| Chr10 | 2921925 | 2926727 |
| Chr10 | 4278944 | 4279262 |
| Chr10 | 5890684 | 5892403 |
| Chr10 | 6387721 | 6388802 |
| Chr10 | 15590591 | 15591243 |
| Chr10 | 19306761 | 19307477 |
| Chr10 | 19830076 | 19843242 |
| Chr11 | 1755307 | 1755955 |
| Chr11 | 1959789 | 1960202 |
| Chr11 | 2705407 | 2705955 |
| Chr11 | 6651969 | 6652734 |
| Chr11 | 15865973 | 15866463 |
| Chr11 | 16225204 | 16226577 |
| Chr11 | 20661228 | 20663355 |
| Chr11 | 20956460 | 20960718 |
| Chr12 | 668869 | 673166 |
| Chr12 | 1798848 | 1820563 |
| Chr12 | 1823026 | 1827565 |
| Chr12 | 2147983 | 2148403 |
| Chr12 | 20326469 | 20327259 |
| Chr12 | 20629047 | 20629601 |
| Chr13 | 137697 | 151926 |
| Chr13 | 588679 | 588830 |
| Chr13 | 723493 | 724669 |
| Chr13 | 1361998 | 1367302 |
| Chr13 | 2340124 | 2344404 |
| Chr13 | 4710267 | 4714114 |
| Chr13 | 6894714 | 6896196 |
| Chr13 | 7085137 | 7090041 |
| Chr13 | 15175479 | 15175841 |
| Chr13 | 15408978 | 15409885 |
| Chr13 | 16523513 | 16523618 |
| Chr14 | 3183985 | 3184420 |
| Chr14 | 3289562 | 3326267 |
| Chr14 | 4073538 | 4074443 |
| Chr14 | 4260530 | 4261574 |
| Chr14 | 4912852 | 4913337 |
| Chr14 | 12981600 | 12982578 |
| Chr14 | 14169447 | 14169811 |
| Chr14 | 15797877 | 15798493 |
| Chr15 | 1534670 | 1535474 |
| Chr15 | 2195911 | 2197545 |
| Chr15 | 2196904 | 2199843 |
| Chr15 | 2204583 | 2211727 |
| Chr15 | 5047364 | 5048079 |
| Chr15 | 5325531 | 5326502 |
| Chr15 | 8713036 | 8715441 |
| Chr15 | 13166124 | 13172156 |
| Chr15 | 13177229 | 13180167 |
| Chr15 | 13277380 | 13286100 |
| Chr15 | 14454314 | 14458584 |
| Chr17 | 1217962 | 1219235 |
| Chr17 | 2618585 | 2619104 |
| Chr17 | 3027812 | 3031108 |
| Chr17 | 6643456 | 6643780 |
| Chr17 | 7370269 | 7371062 |
| Chr17 | 7642794 | 7644652 |
| Chr18 | 3065264 | 3066469 |
| Chr18 | 3185082 | 3185772 |
| Chr18 | 3365299 | 3365718 |
| Chr18 | 3465939 | 3465976 |
| Chr18 | 4169135 | 4171987 |
| Chr18 | 4332602 | 4345781 |
| Chr18 | 5197165 | 5198922 |
| Chr18 | 10476317 | 10476755 |
| Chr18 | 11193291 | 11193822 |
| Chr18 | 11905011 | 11906525 |
| Chr19 | 5514285 | 5516236 |
| Chr19 | 6391518 | 6391642 |
| Chr19 | 8507087 | 8507770 |
| Chr20 | 2565928 | 2570356 |
| Chr20 | 3325544 | 3326577 |
| Chr20 | 6419791 | 6421806 |
| Chr20 | 8683335 | 8684260 |
| Chr20 | 9194889 | 9199820 |
| Chr20 | 14045862 | 14047959 |
| Chr21 | 758527 | 763870 |
| Chr21 | 1744895 | 1745568 |
| Chr21 | 2554911 | 2555452 |
| Chr21 | 4698306 | 4701041 |
| Chr21 | 5699304 | 5700693 |
| Chr21 | 6604755 | 6606787 |
| Chr22 | 1256089 | 1412083 |
| Chr22 | 2354235 | 2354986 |
| Chr22 | 3742455 | 3745778 |
| Chr22 | 4020340 | 4039955 |
| Chr22 | 4645349 | 4648629 |
| Chr22 | 5303807 | 5304783 |
| Chr23 | 2550045 | 2552180 |
| Chr23 | 2829674 | 2833745 |
| Chr23 | 3286189 | 3289241 |
| Chr23 | 5222503 | 5224228 |
| Chr23 | 6258013 | 6259182 |
| Chr23 | 6324682 | 6325384 |
| Chr24 | 1493513 | 1496480 |
| Chr24 | 1865062 | 1865645 |
| Chr24 | 3286057 | 3286122 |
| Chr24 | 4804470 | 4812649 |
| Chr24 | 5827653 | 5827981 |
| Chr24 | 6336446 | 6338894 |
| Chr25 | 873183 | 874492 |
| Chr25 | 907468 | 917705 |
| Chr25 | 1028449 | 1062584 |
| Chr25 | 1030731 | 1111618 |
| Chr25 | 1030731 | 1068005 |
| Chr25 | 2106646 | 2115576 |
| Chr26 | 2373581 | 2377770 |
| Chr26 | 2631418 | 2632321 |
| Chr26 | 2949620 | 2959702 |
| Chr26 | 3316796 | 3517666 |
| Chr26 | 3383127 | 3519540 |
| Chr26 | 4447276 | 4450715 |
| Chr26 | 5183395 | 5184365 |
| Chr27 | 498437 | 498654 |
| Chr27 | 523751 | 523847 |
| Chr27 | 4320521 | 4337530 |
| Chr27 | 4320521 | 4333693 |
| Chr27 | 4320521 | 4334897 |
| Chr27 | 4320608 | 4333693 |
| Chr27 | 4320608 | 4334878 |
| Chr27 | 4903014 | 4910391 |
| Chr27 | 4915137 | 4949926 |
| Chr28 | 3409393 | 3409629 |
| Chr28 | 3993440 | 4022667 |
| Chr28 | 3993440 | 4077070 |
| Chr28 | 4137043 | 4143391 |
| Chr28 | 4508974 | 4510270 |
| Chr28 | 4783080 | 4794852 |
| ChrLGE22 | 120962 | 143294 |
| ChrLGE22 | 291580 | 343964 |
| ChrLGE22 | 1148805 | 1161256 |
| ChrLGE22 | 1240603 | 1257948 |
| ChrLGE22 | 1475633 | 1499098 |
| ChrLGE22 | 1654313 | 1656685 |
| ChrZ | 122857 | 128300 |
| ChrZ | 157855 | 158030 |
| ChrZ | 10025720 | 10027416 |
| ChrZ | 15509231 | 15510410 |
| ChrZ | 19233617 | 19234224 |
| ChrZ | 27019073 | 27019651 |
| ChrZ | 27667295 | 27672705 |
| ChrZ | 28624579 | 28627949 |
| ChrZ | 37262751 | 37265832 |
| ChrZ | 38577951 | 38587828 |
| ChrZ | 43251840 | 43253122 |
| ChrZ | 50595606 | 50608021 |
| ChrZ | 50595606 | 50608021 |
| ChrZ | 53997604 | 54032629 |
| ChrZ | 53997604 | 54032629 |
| ChrZ | 56298755 | 56300592 |
| ChrZ | 57504859 | 57534814 |
| ChrZ | 57590287 | 57600692 |
| ChrZ | 57891362 | 57895876 |
| ChrZ | 59297876 | 59298175 |
