# Supplementary material for: High-Resolution Mapping of Crossover and Non-crossover Recombination Events by Whole-Genome Re-sequencing of an Avian Pedigree
Source: PLoS Genet. 2016 May 24;12(5):e1006044. doi: 10.1371/journal.pgen.1006044 (PMC4878770; doi:10.1371/journal.pgen.1006044)
Supplement: S3 Table — (DOCX) [file pgen.1006044.s003.docx]

**Supplementary Table 3**.

| Offspring ID | Maternal | | Paternal | |
| --- | --- | --- | --- | --- |
|  | No events | Median length (bp) | No events | Median length (bp) |
| CS4 | 22 | 2,847 | 41 | 1,193 |
| CS5 | 16 | 1,698 | 43 | 1,837 |
| CS6 | 29 | 1,389 | 42 | 2,185 |
| CS7 | 29 | 2,476 | 41 | 1,373 |
| CS9 | 25 | 1,286 | 37 | 1,696 |
