# Supplementary material for: High-Resolution Mapping of Crossover and Non-crossover Recombination Events by Whole-Genome Re-sequencing of an Avian Pedigree
Source: PLoS Genet. 2016 May 24;12(5):e1006044. doi: 10.1371/journal.pgen.1006044 (PMC4878770; doi:10.1371/journal.pgen.1006044)
Supplement: S4 Table — (DOCX) [file pgen.1006044.s004.docx]

**Supplementary Table 4.**

| Chromosome | Position of NCO SNP |
| --- | --- |
| Chr1 | 1457106 |
| Chr1 | 2942875 |
| Chr1 | 10970531 |
| Chr1 | 27055789 |
| Chr1 | 33999208 |
| Chr1 | 34273010 |
| Chr1 | 47058571 |
| Chr1 | 49742049 |
| Chr1 | 50539362 |
| Chr1 | 56317310 |
| Chr1 | 63007810 |
| Chr1 | 70342692 |
| Chr1 | 80859842 |
| Chr1 | 90408214 |
| Chr1 | 91424688 |
| Chr1 | 97921148 |
| Chr1A | 153301 |
| Chr1A | 781559 |
| Chr1A | 2303173 |
| Chr1A | 28409616 |
| Chr1A | 31350776 |
| Chr1A | 35377366 |
| Chr1A | 46432873 |
| Chr1A | 48453861 |
| Chr1A | 52361938 |
| Chr1A | 69403861 |
| Chr1A | 72857450 |
| Chr1A | 74055289 |
| Chr2 | 15992517 |
| Chr2 | 24657954 |
| Chr2 | 39161449 |
| Chr2 | 39306704 |
| Chr2 | 46194511 |
| Chr2 | 59512536 |
| Chr2 | 75971669 |
| Chr2 | 79675713 |
| Chr2 | 81754552 |
| Chr2 | 97430681 |
| Chr2 | 100960596 |
| Chr2 | 103508114 |
| Chr2 | 104327887 |
| Chr2 | 109342186 |
| Chr2 | 117606135 |
| Chr2 | 121263606 |
| Chr2 | 122990325 |
| Chr2 | 129204161 |
| Chr2 | 137909191 |
| Chr2 | 144947697 |
| Chr2 | 145430989 |
| Chr2 | 157332336 |
| Chr2 | 157357322 |
| Chr3 | 10657833 |
| Chr3 | 12659018 |
| Chr3 | 12676076 |
| Chr3 | 17428081 |
| Chr3 | 22313449 |
| Chr3 | 35216269 |
| Chr3 | 43101759 |
| Chr3 | 47761045 |
| Chr3 | 48877204 |
| Chr3 | 66322014 |
| Chr3 | 67734783 |
| Chr3 | 73118157 |
| Chr3 | 73547525 |
| Chr3 | 88460013 |
| Chr3 | 93949612 |
| Chr3 | 103332216 |
| Chr3 | 110885494 |
| Chr3 | 111243391 |
| Chr3 | 113323200 |
| Chr4 | 10917636 |
| Chr4 | 28156034 |
| Chr4 | 28156034 |
| Chr4 | 33411911 |
| Chr4 | 36646471 |
| Chr4 | 43506650 |
| Chr4 | 48730606 |
| Chr4 | 51140601 |
| Chr4 | 69884220 |
| Chr4A | 1184931 |
| Chr4A | 1196872 |
| Chr4A | 3246321 |
| Chr4A | 5184012 |
| Chr4A | 7415904 |
| Chr4A | 11706455 |
| Chr4A | 16347802 |
| Chr4A | 17060991 |
| Chr5 | 210772 |
| Chr5 | 1259256 |
| Chr5 | 1810856 |
| Chr5 | 5844458 |
| Chr5 | 5857942 |
| Chr5 | 5873625 |
| Chr5 | 11297510 |
| Chr5 | 11699286 |
| Chr5 | 20877050 |
| Chr5 | 34532663 |
| Chr5 | 36124168 |
| Chr5 | 47364201 |
| Chr5 | 51761149 |
| Chr5 | 58282862 |
| Chr5 | 63001147 |
| Chr5 | 63718290 |
| Chr5 | 63918293 |
| Chr5 | 64474439 |
| Chr6 | 6867562 |
| Chr6 | 6982589 |
| Chr6 | 22052594 |
| Chr6 | 23403044 |
| Chr7 | 11172796 |
| Chr7 | 24162598 |
| Chr7 | 24422942 |
| Chr7 | 26263618 |
| Chr7 | 37351853 |
| Chr8 | 4829065 |
| Chr8 | 6499062 |
| Chr8 | 10743559 |
| Chr8 | 21927767 |
| Chr8 | 26128288 |
| Chr8 | 29492830 |
| Chr8 | 31934664 |
| Chr9 | 10250071 |
| Chr10 | 10438642 |
| Chr10 | 15166361 |
| Chr10 | 18457032 |
| Chr10 | 19730217 |
| Chr11 | 1490212 |
| Chr12 | 2601301 |
| Chr12 | 4030016 |
| Chr12 | 21060188 |
| Chr13 | 6783788 |
| Chr13 | 16294990 |
| Chr14 | 4056004 |
| Chr14 | 15408085 |
| Chr15 | 836441 |
| Chr15 | 2211187 |
| Chr17 | 8546207 |
| Chr17 | 11285025 |
| Chr20 | 1798326 |
| Chr20 | 1927770 |
| Chr20 | 5153538 |
| Chr20 | 8102761 |
| Chr20 | 14345357 |
| Chr21 | 1246937 |
| Chr22 | 4099641 |
| Chr23 | 1323374 |
| Chr23 | 1617698 |
| Chr23 | 2004781 |
| Chr23 | 6833653 |
| Chr24 | 1573492 |
| Chr25 | 2107392 |
| Chr26 | 1997256 |
| Chr26 | 4852085 |
| Chr27 | 506915 |
| Chr28 | 4424616 |
| ChrZ | 21399733 |
