# Supplementary figures and images for: High-Resolution Mapping of Crossover and Non-crossover Recombination Events by Whole-Genome Re-sequencing of an Avian Pedigree
Source: PLoS Genet. 2016 May 24;12(5):e1006044. doi: 10.1371/journal.pgen.1006044 (PMC4878770; doi:10.1371/journal.pgen.1006044)

**Supplementary Fig 1.**

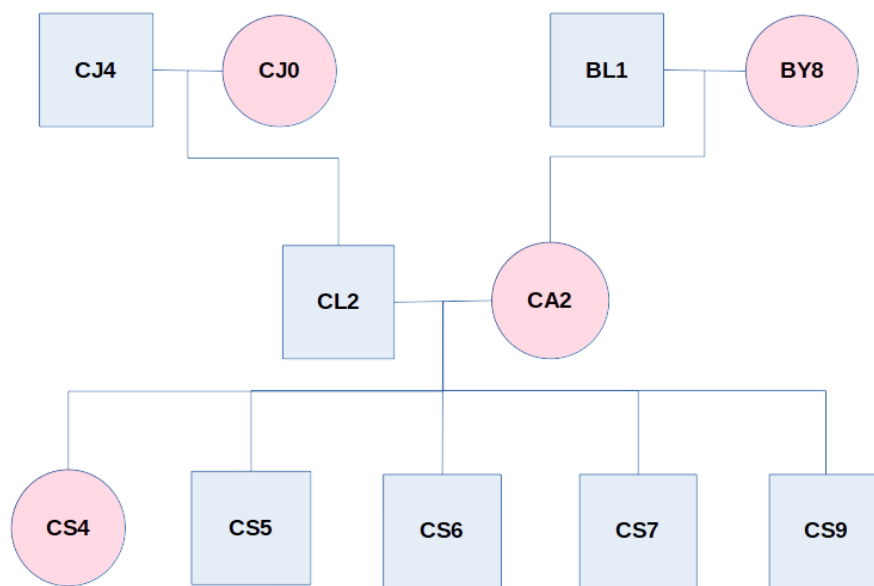

Supplement: S1 Fig — Only regions shorter than 20 kb are shown; there are 24 additional intervals larger than 20 kb. (PDF) [file pgen.1006044.s005.pdf]

Supplementary Fig 2.

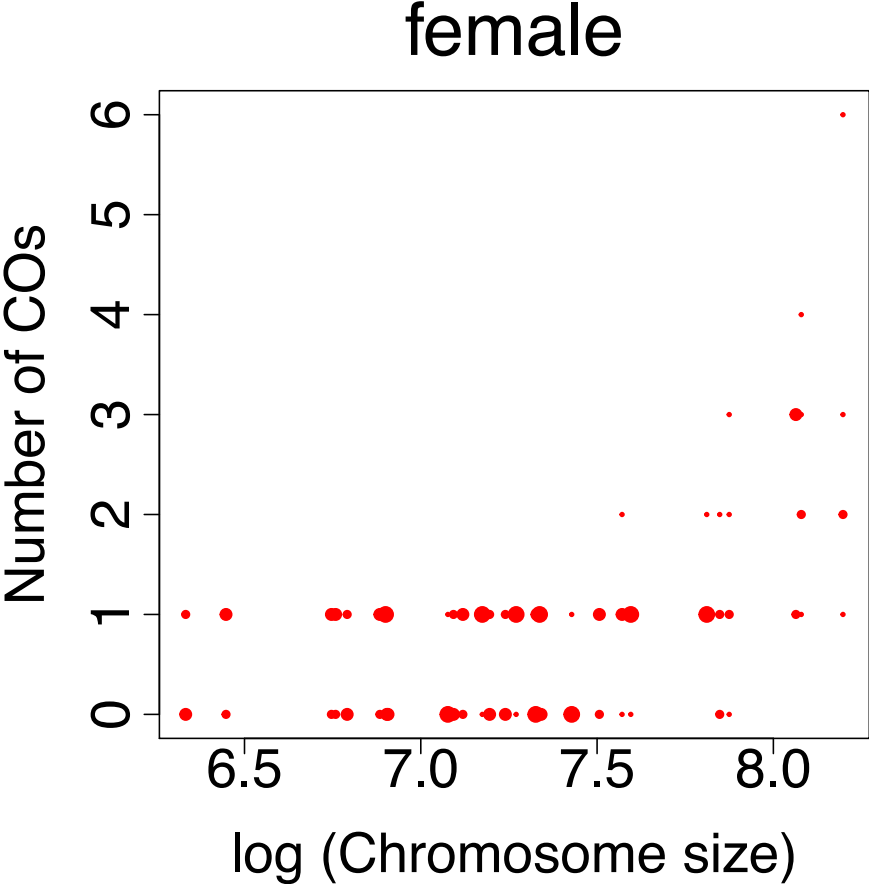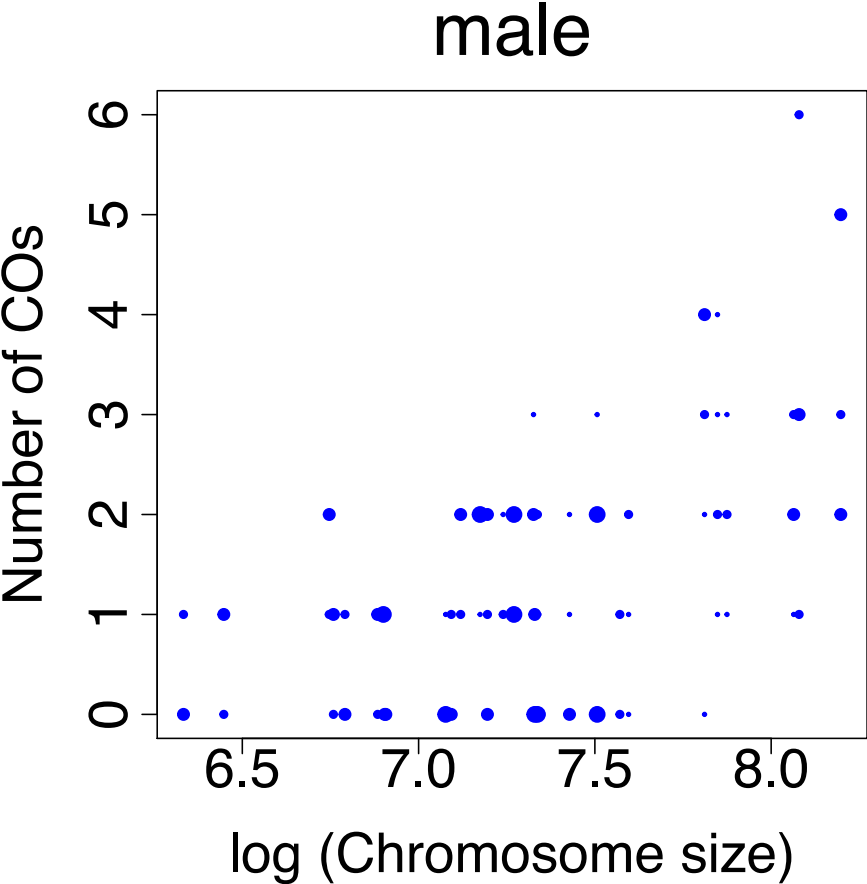

Supplement: S2 Fig — (PDF) [file pgen.1006044.s006.pdf]

**Supplementary Fig 3.**

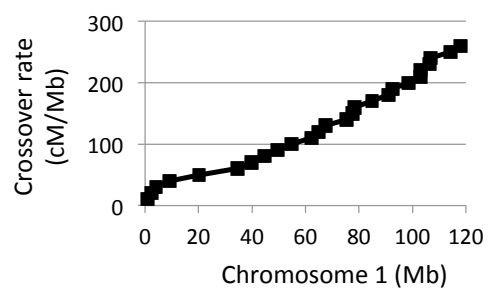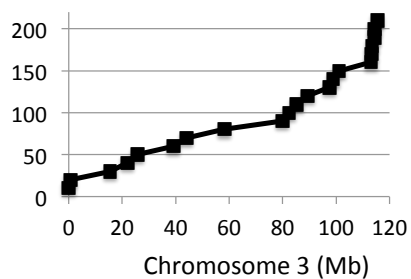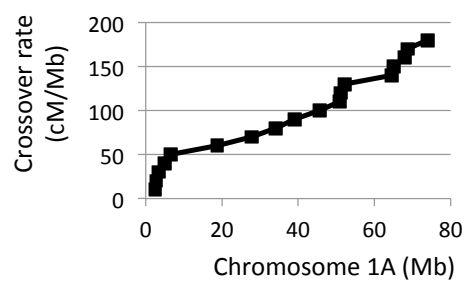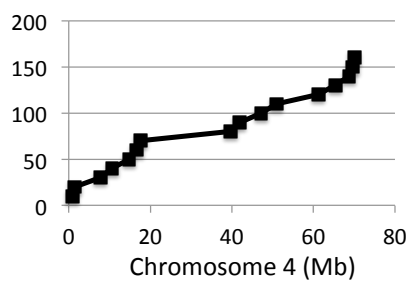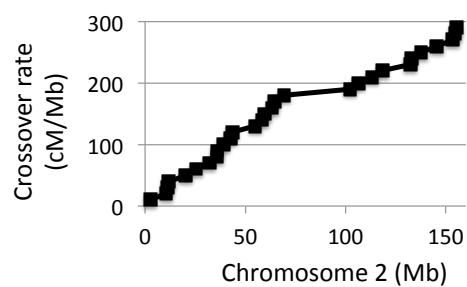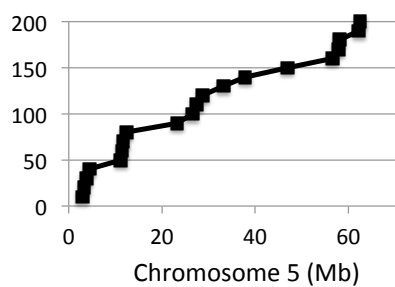

Supplement: S3 Fig — Cumulative, sex-average genetic distance along chromosomes obtained from the distribution of CO events are shown for the six largest chromosomes. (PDF) [file pgen.1006044.s007.pdf]

**Supplementary Fig 4.**

**a)**

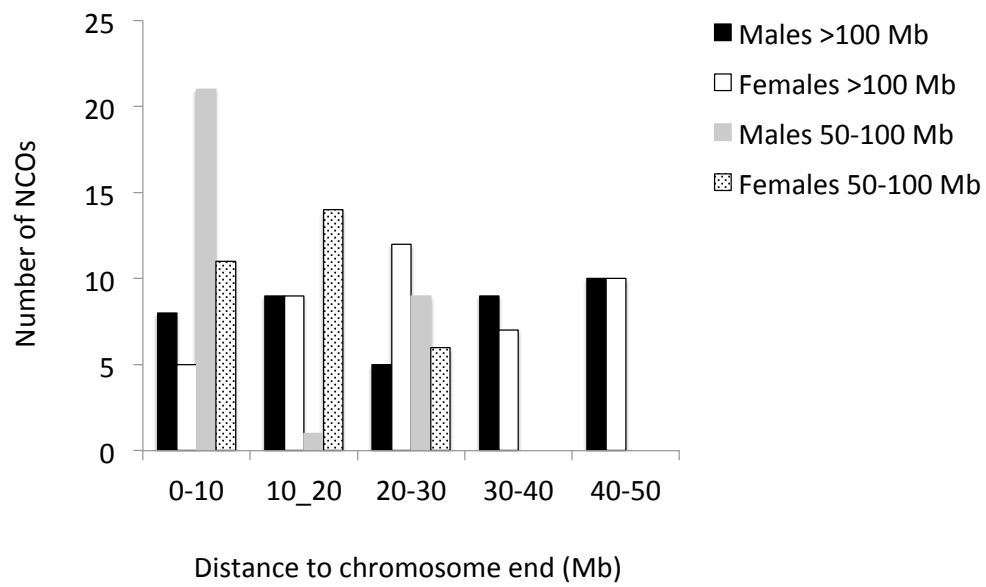

**b)**

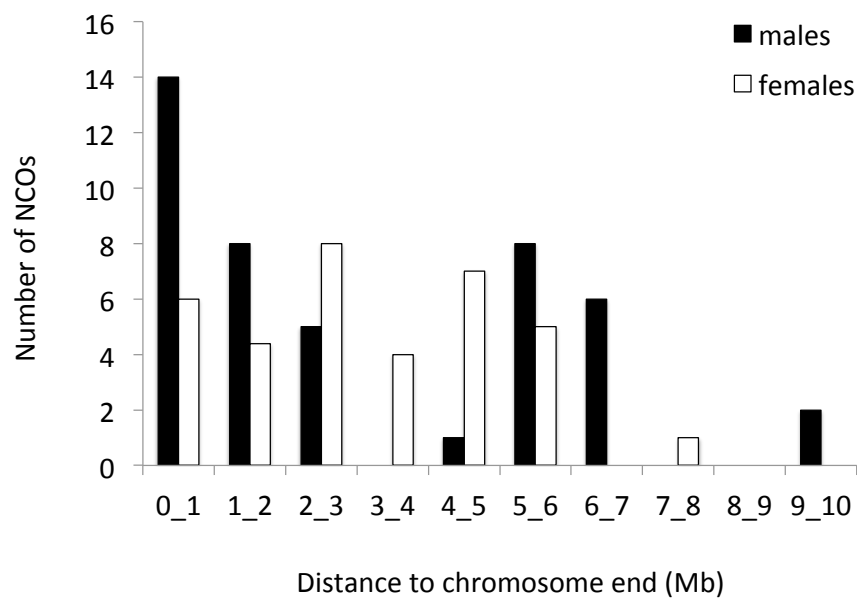

Supplement: S4 Fig — (a) chromosomes >100 Mb (males, black; female, white) and chromosomes 50–100 Mb (males, grey; females dotted) for 10 Mb intervals, and (b) the terminal 10 Mb of all chromosomes in 1 Mb intervals (males, black; female, white). (PDF) [file pgen.1006044.s008.pdf]

Supplementary Fig 5.

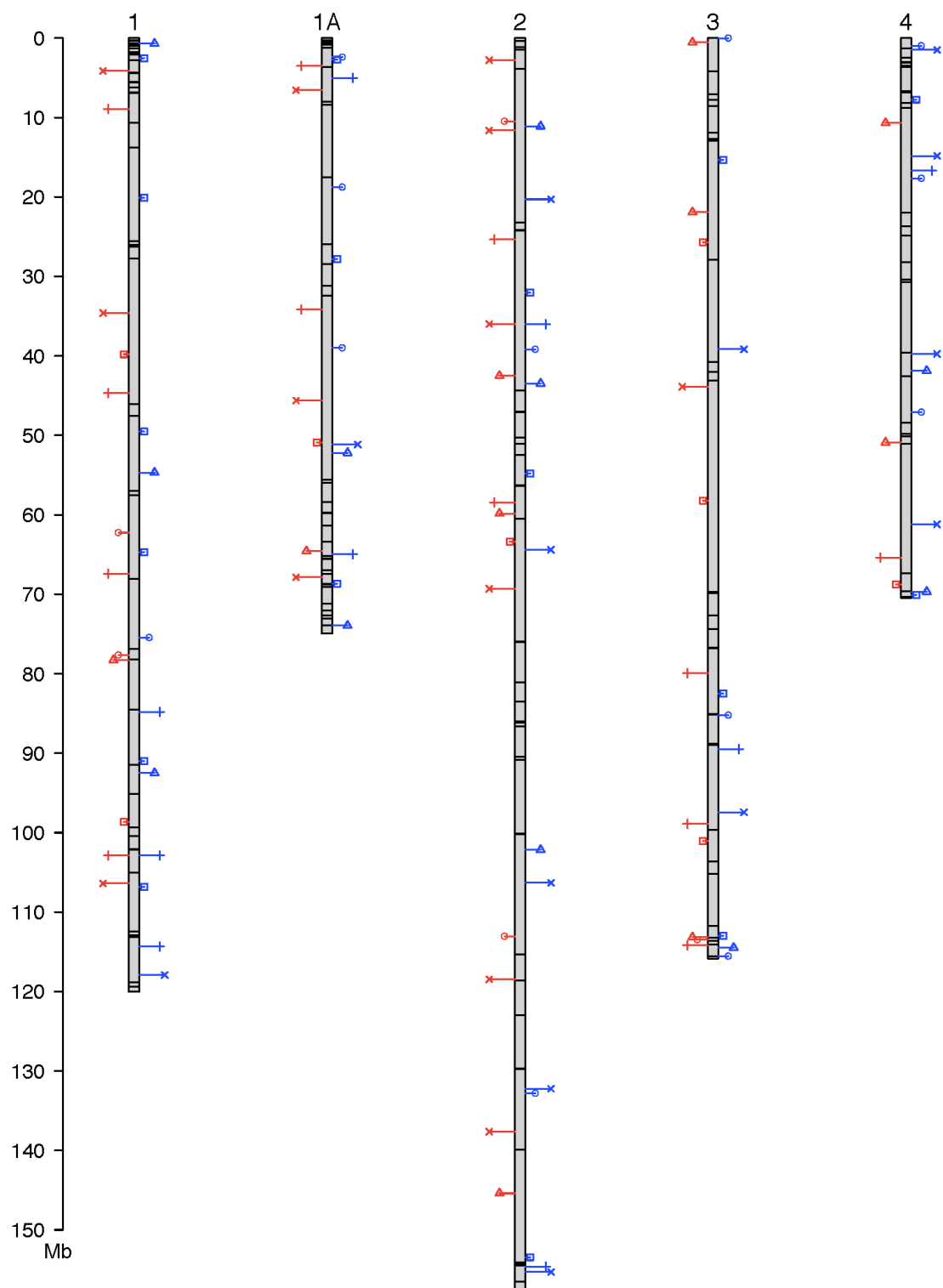

Supplement: S5 Fig — Maternal events are shown to the left of each chromosomes in red and paternal events are shown to the right in blue. Each symbol (diamond, triangle, box, cross, plus) represents one of the five F2 offspring in which CO events were observed. (PDF) [file pgen.1006044.s009.pdf]

**Supplementary Fig 6.**

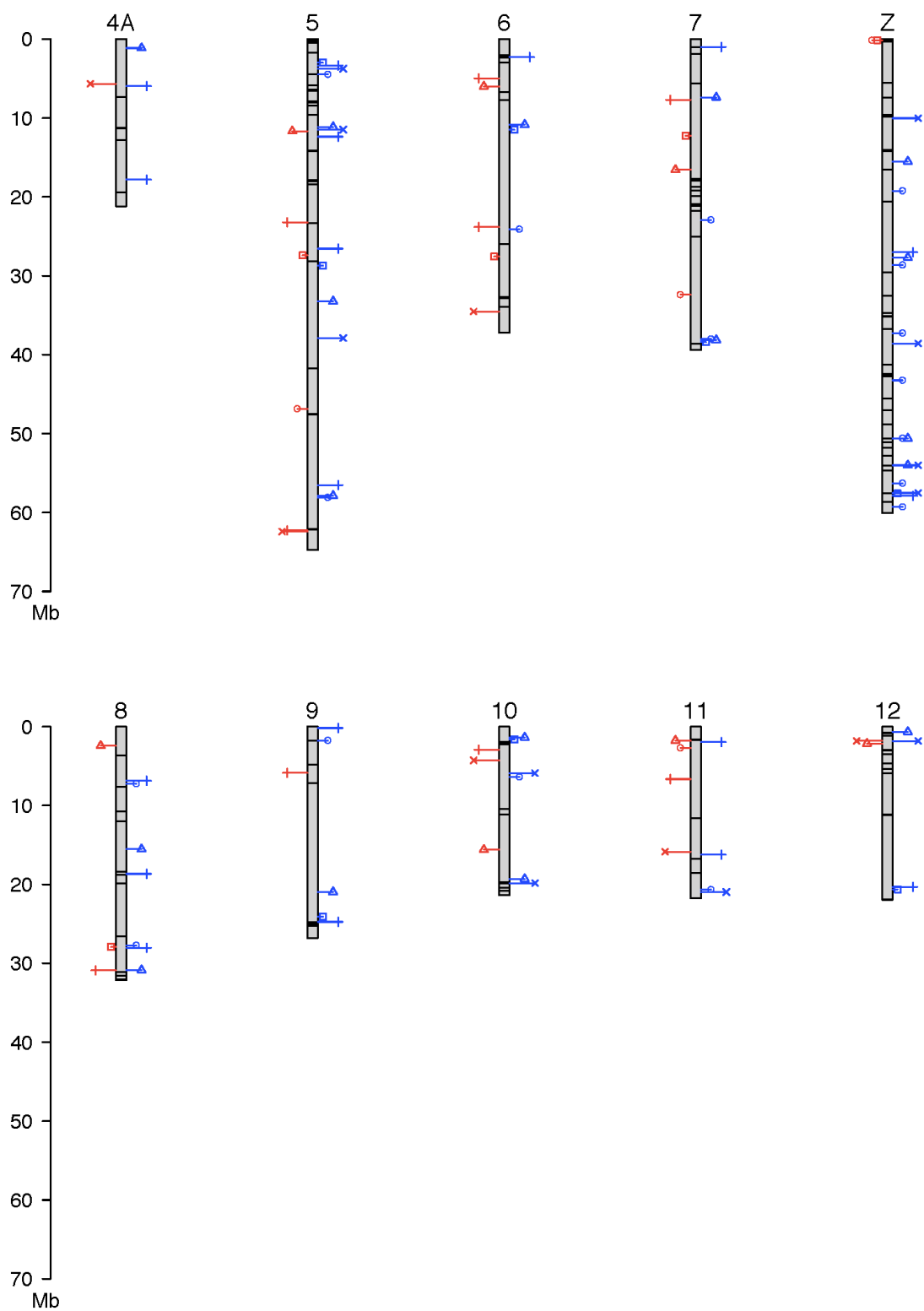

Supplement: S6 Fig — Maternal events are shown to the left of each chromosomes in red and paternal events are shown to the right in blue. Each symbol (diamond, triangle, box, cross, plus) represents one of the five F2 offspring in which CO events were observed. (PDF) [file pgen.1006044.s010.pdf]

**Supplementary Fig 7.**

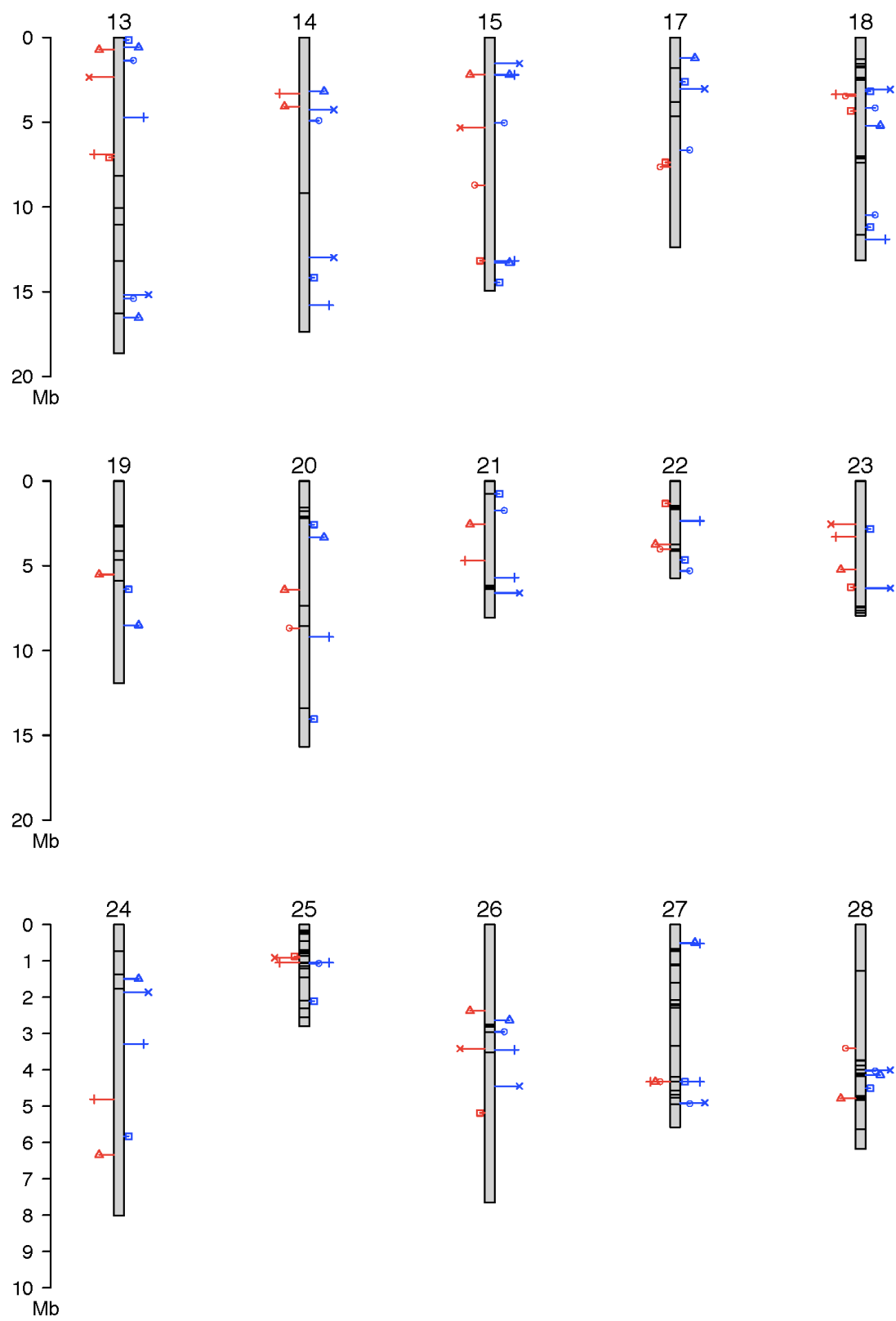

Supplement: S7 Fig — Maternal events are shown to the left of each chromosomes in red and paternal events are shown to the right in blue. Each symbol (diamond, triangle, box, cross, plus) represents one of the five F2 offspring in which CO events were observed. (PDF) [file pgen.1006044.s011.pdf]

Supplementary Fig 8.

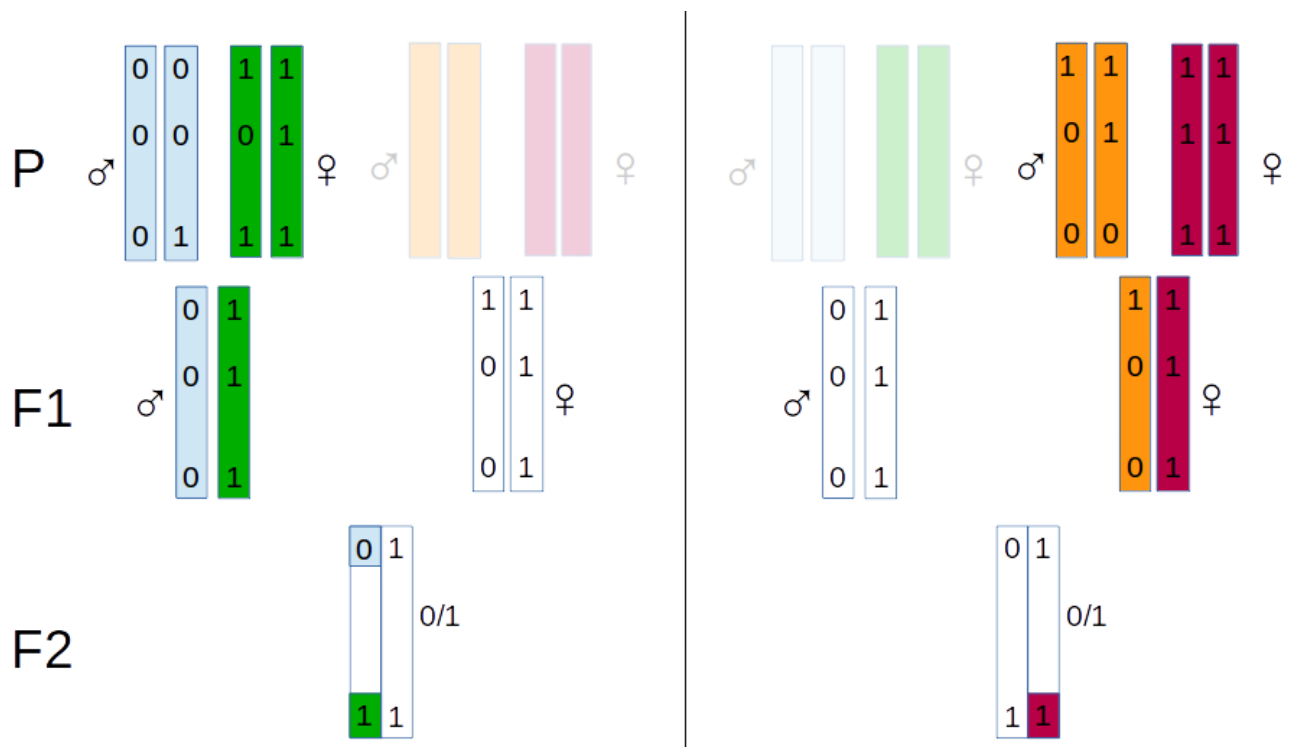

Supplement: S8 Fig — Phasing of haplotypes originating from the paternal (left) and maternal (right) grandparents. Genotypes are shown only for the five individuals that need to be considered for the line in question, and are given as 0 (reference allele) and 1 (alternative allele). In the paternal example, the first and third SNPs in the F2 are informative while the second SNP is uninformative because both 0 and 1 can come from either F1 parent. In the maternal example, only the third SNP in the F2 can be traced back to the P generation and is informative. The first SNP can be traced back to the F1 but is not informative. (PDF) [file pgen.1006044.s012.pdf]
